# Universal Interfacial Engineering via Amorphous Inorganic Binders: Passivating Surface States and Accelerating Hole Transfer across Metal Oxide Photoanodes in Photoelectrochemical Water Oxidation

**DOI:** 10.1021/acs.jpclett.6c00767

**Published:** 2026-04-17

**Authors:** Po-Keng Hsiao, Yen-Lun Kung, Yun-Pei Liu, Shih-Wen Tseng, Tetsu Yonezawa, Chun-Hu Chen, Ying-Chih Pu

**Affiliations:** † Department of Materials Science, 33561 National University of Tainan, Tainan 700301, Taiwan; ‡ Department of Chemistry, National Sun Yat-sen University, Kaohsiung 80424, Taiwan; § Core Facility Center of National Cheng Kung University, Tainan 70101, Taiwan; ∥ Division of Materials Science and Engineering, Faculty of Engineering, 12810 Hokkaido University, Hokkaido 060-8628, Japan; ⊥ Department of Chemical Engineering, Faculty of Engineering, Chulalongkorn University, Phayathai Road, Pathumwan, Bangkok 10330, Thailand; # Green Hydrogen Research Center, National Sun Yat-sen University, Kaohsiung 80424, Taiwan

## Abstract

Interfacial engineering of photoanodes is essential for efficient solar water splitting; however, many existing strategies rely on complex, multistep processes or hydrothermal growth, limiting scalability and reproducibility. Here, we present a universal interfacial engineering platform based on a sub-2 min acidic redox-assisted deposition (ARD) of an amorphous inorganic binder, cobalt–manganese oxyhydroxide (CMOH). Using two-step-fabricated BiVO_4_ as a literature-standard benchmark, we demonstrate that an ultrathin CMOH overlayer markedly enhances photoelectrochemical (PEC) water oxidation performance. The conformal CMOH coating (∼3.5 nm) is deposited under ambient conditions via a simple dip-coating process and is broadly applicable to metal oxide photoanodes, including BiVO_4_, WO_3_, and ZnO. The CMOH-modified BiVO_4_ photoanode delivers a high photocurrent density of ∼6.0 mA cm^–2^ and a hole-transfer efficiency of ∼82% at 1.23 V_RHE_, together with a remarkable applied bias photon-to-current efficiency (ABPE) of 1.68% at 0.66 V_RHE_. These enhancements originate from effective surface-state passivation by the CMOH overlayer, leading to over a 4-fold reduction in the surface recombination rate constant, as revealed by intensity-modulated photocurrent spectroscopy (IMPS). In addition, the reduced Tafel slope (78.5 mV dec^–1^) suggests that the CMOH layer promotes interfacial charge transfer and enhances the water oxidation kinetics of the BiVO_4_ photoanode. This work elucidates the critical role of amorphous CMOH oxygen evolution catalysts in mediating photoinduced charge-carrier dynamics and establishes a facile and scalable strategy to broadly enhance the PEC performance of metal oxide photoanodes for large-scale solar fuel production.

## Graphical abstract

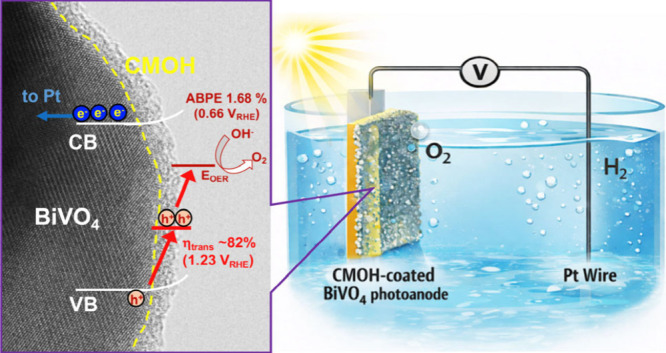

Developing efficient and robust photoelectrochemical (PEC) water oxidation systems is a critical step toward sustainable hydrogen production.^1^ While PEC water splitting offers a clean route to convert solar energy into chemical fuels, simultaneously achieving high energy conversion efficiency and long-term operational stability—particularly on large-area electrodes required for industrial deployment—remains a major challenge. In most PEC systems, the photoanode represents the primary performance bottleneck due to the intrinsically sluggish, multielectron oxygen evolution reaction (OER) and the poor durability of semiconductor surfaces under prolonged illumination and anodic bias.^2^ These limitations are further exacerbated when extending photoanode architectures from laboratory-scale devices to large-area configurations, where uniform charge transport, interfacial stability, and scalable fabrication become critical concerns.^3,4^ Consequently, the development of high-performance, scalable photoanodes that can reliably match advanced photocathodes is essential for realizing practically viable PEC water splitting systems.

Among candidate materials, bismuth vanadate (BiVO_4_) has emerged as one of the most promising photoanodes for PEC water oxidation owing to its suitable bandgap (∼2.4–2.5 eV), visible-light absorption, and high theoretical photocurrent density (∼7.5 mA cm^–2^ under AM 1.5G).^5^ However, practical BiVO_4_ photoanodes suffer from inefficient hole transfer at the semiconductor–electrolyte interface, where surface states and defect-associated traps promote interfacial recombination and suppress charge injection.^6–8^ These interfacial losses are particularly severe in BiVO_4_, where shallow surface traps and accumulated holes dominate performance degradation. In addition, BiVO_4_ is prone to photocorrosion during anodic operation, as vanadium species can leach from the lattice under oxidative bias.^9^ Unlike purely electrochemical systems, photoanode performance is governed not only by catalytic activity but also by the efficiency of photogenerated hole extraction, suppression of surface-state-mediated recombination, and resistance to photoinduced corrosion under sustained illumination.^10,11^ Accordingly, surface modification with appropriate oxygen evolution catalysts (OECs) has become a key strategy to enhance both activity and stability of BiVO_4_ photoanodes.^12^

Significant progress has been made by coupling BiVO_4_ with various OECs that function as hole-transfer layers.^13^ For example, FeOOH/NiOOH overlayers deposited via alternating photoelectrodeposition have been shown to markedly accelerate water oxidation kinetics and improve stability.^14^ NiCo layered double hydroxides deposited cathodically have also been reported to enhance photon-to-current conversion efficiency by improving interfacial charge transfer and separation.^15^ More recently, hydrothermally grown NiFe metal–organic framework (MOF) layers have demonstrated dual roles as protective coatings and cocatalysts, albeit requiring elevated temperatures and extended processing times.^16^ Notably, when OECs are coupled to BiVO_4_ photoanodes, their function extends beyond conventional electrocatalysis and becomes intrinsically coupled to photoinduced charge-carrier dynamics, governing charge separation, transfer, and recombination at the interface.^13^ Despite these advances, most reported fabrication strategies rely on multistep electrochemical, thermal, or pressurized processes with long processing times,^5,10,17^ posing challenges for reproducibility, uniformity, and scalability—particularly for large-area applications. Thus, a facile, solution-processable, and scalable approach for the integration of an OEC is highly desirable.

In our previous work, we developed a simple and effective aqueous-phase strategy known as acidic redox-assisted deposition (ARD),^18^ and applied it to a family of earth-abundant multimetal (oxy)­hydroxide coatings,^19^ with cobalt–manganese oxyhydroxide (CMOH) as a representative system.^20–22^ This solution-based method proceeds under mild conditions—typically ambient temperature or gentle heating—and has been demonstrated to be compatible with scalable fabrication, including roll-to-roll processing.^23^ The ARD process yields ultrathin (∼6–9 nm), amorphous, and fully conformal coatings on a wide range of substrates, including metals, ceramics, and polymers.^18^ Importantly, such ultrathin ARD products exhibit high optical transparency (up to ∼ 98% transmittance),^24^ making them suitable as cocatalyst/overlayer films that preserve light delivery to the underlying photoabsorber. Furthermore, all these ARD-produced films possess strong adhesion and robust mechanical durability, which are critical for preventing catalyst delamination or peel-off during prolonged OER operation.^25^ Yet this facile ARD process has not been explored to decorate CMOH on BiVO_4_ photoanodes to serve as protective layers or activity promoters for PEC water oxidation. The effects of CMOH on interfacial charge carrier dynamics of BiVO_4_ photoanodes in PEC still remain deficient even now.

In this work, the solution-processable ARD method to decorate BiVO_4_ photoanodes with a CMOH OEC layer by the dip-coating process with 1–16 min under normal ambient conditions is illustrated as Scheme 1. The morphology, structure, and interfacial chemical states of the CMOH-coated BiVO_4_ photoanodes were characterized by microscopy and spectroscopy. The PEC performance and activity enhancement of the CMOH-modified BiVO_4_ photoanodes were systematically investigated. In addition, intensity-modulated photocurrent spectroscopy (IMPS) was conducted to elucidate the charge-transfer and recombination kinetics of photogenerated carriers during PEC water oxidation. The insights gained from this study reveal a previously unexplored role of amorphous oxyhydroxide layers in governing interfacial charge-transfer and recombination dynamics at semiconductor photoanodes, establishing design principles for stabilizing oxide photoanodes via ultrathin amorphous redox-active interfaces.

**Scheme 1:**
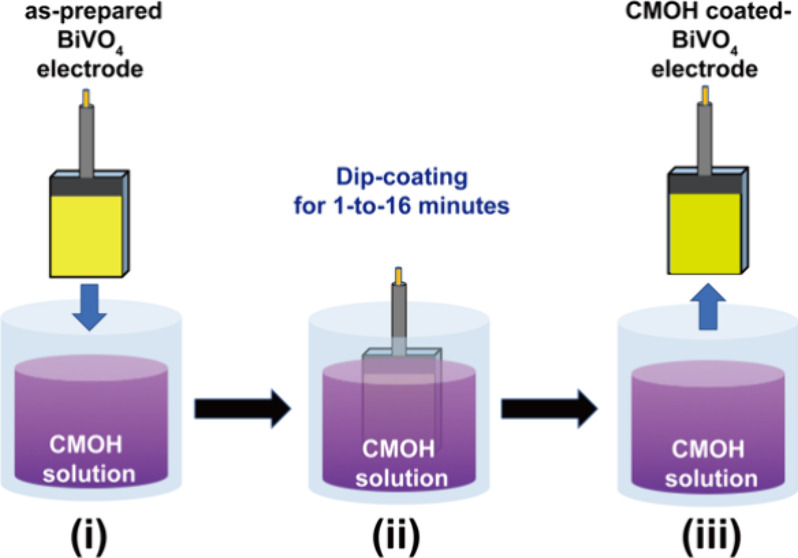
Schematic Illustration of the Solution-Processable ARD Method to Decorate CMOH Layer on BVO Photoanodes within 1–16 min under Normal Ambient Conditions: (i) As-Prepared ARD Precursor Solution and BiVO_4_ Electrode; (ii) Immersion of BiVO_4_ Electrode into the Precursor Solution for 1–16 min for CMOH Deposition; (iii) After Taking Out and Cleaning, the CMOH-Coated BiVO_4_ Electrode Was Obtained for PEC Utilization

ARD technique enables the spontaneous formation of CMOH films through a solution-phase redox reaction between Co^2+^ ions and permanganate (MnO_4_^–^) under mildly acidic conditions. This redox couple facilitates the products comprised of Co­(III/IV) and Mn­(III/IV) hydroxide species in a single step without requiring thermal or electrochemical activation, as in the equation

1 $$9{\mathrm{Co}}^{2+}\left(\mathrm{aq}\right)+3{{\mathrm{MnO}}_{4}}^{-}\left(\mathrm{aq}\right)+14H_{2}O\left(l\right)\to {\mathrm{Co}}_{9}{\mathrm{Mn}}_{3}O_{26}H_{13}\left(s\right)+15H^{+}\left(\mathrm{aq}\right)$$

where Co_9_Mn_3_O_26_H_13_ is the typical chemical formula of CMOH.^26^ The generation of H^+^ during the synthesis lowers the solution pH to around 2–3, distinguishing that the ARD products do not come from the conventional base-precipitation route. Importantly, the deposition process is time-dependent: a longer exposure results in thicker films. These manganese species are known to primarily enable the strong adhesion for CMOH to bind on the arbitrary surfaces (e.g., metal, ceramic, and organic surfaces),^20,24,27^ applicable to the surfaces of BiVO_4_, WO_3_, and ZnO in this work.

Figure 1a shows SEM and TEM images of the BiVO_4_ samples after post-CMOH deposition. For example, the sample notation “BVO_CMOH-*x*m” denotes a BiVO_4_ electrode treated with *x*-min CMOH deposition, where the tested deposition time in this work ranges from 1 to 16 min (*x* = 1, 2, 4, 8 and 16). The SEM and TEM images of Bare BVO are also shown in Figure S1 for comparison. All the samples present a coral-like morphology with nanopores. After the CMOH deposition, additional deposition on the surface of BiVO_4_ can be observed, while the surface roughness was significantly enhanced. When the decoration time was longer than 4 min, particle formation on the BiVO_4_ surface was clearly observed. This agrees with the partial nucleation that does occur on the surface of BiVO_4_,^28^ and the studies show the film products and powder products are mostly the same in composition.^26^ The amorphous features of the conformal CMOH coating on the surface of BiVO_4_ can be clearly observed for these samples, agreeing well with our previous research.^18^ High reproducibility can be achieved through the precise control of the deposition time. The histogram of the thickness distribution for these amorphous layers is shown in Figure S2. The average thickness for the samples has been estimated and is summarized in Table 1. Significantly, the thickness of the amorphous layer on the surface of BiVO_4_ can be well controlled by increasing the decoration period in the post-ARD process. Nevertheless, the thickness distribution would become wider as the decoration period reached 8 and 16 min, which is consistent with the observed particle aggregation on the BiVO_4_ surface in SEM characterization.

**Figure 1:**
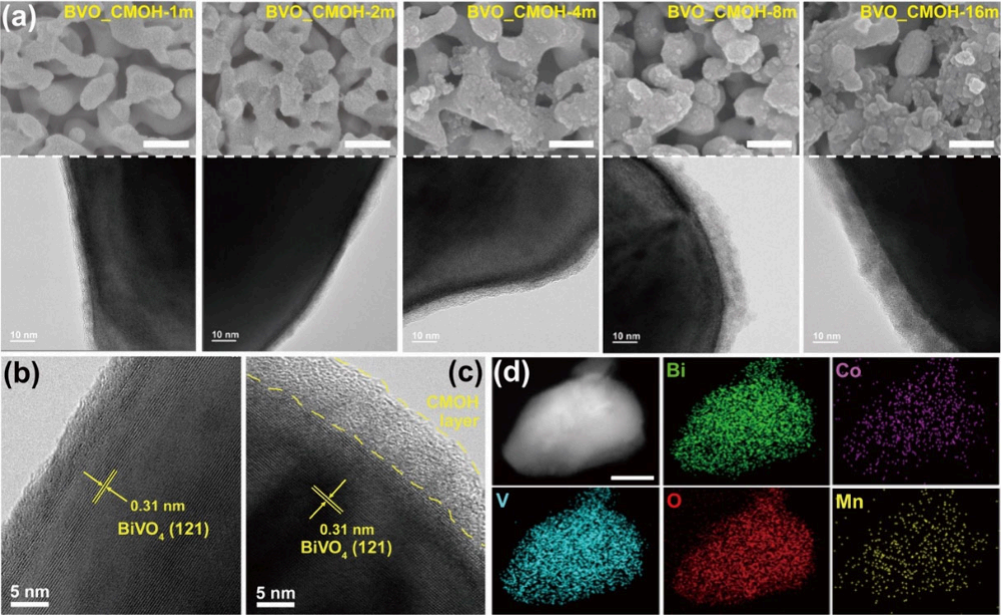
(a) SEM (upper panel) and TEM (bottom panel) images of BVO_CMOH-1m, BVO_CMOH-2m, BVO_CMOH-4m, BVO_CMOH-8m and BVO_CMOH-16m (the scale bars of SEM images are 250 nm). HR-TEM images of (b) Bare BVO and (c) BVO_CMOH-16m. (d) HAADF-STEM image and DES elemental mapping of BVO_CMOH-16m (the scale bar is 50 nm).

**Table 1:** Thickness of CMOH Layer and PEC Performance of Bare BVO and BVO Samples with Post-ARD Process

	CMOH Thickness (nm)	Photocurrent Density @ 1.23 V_RHE_ (mA/cm^2^)	IPCE at 450 nm @ 1.23 V_RHE_ (%)	ABPE (%)	ABPE (V_RHE_)	η_trans_ @ 1.23 V_RHE_ (%)	η_sep_ @ 1.23 V_RHE_ (%)	C_dl_ (μF/cm^2^)
Bare BVO	0	2.2	27.7	0.37	0.89	31.5	62.9	4.36
BVO_CMOH-1m	2.0 ± 0.5	5.5	81.3	1.61	0.66	79.6	62.3	8.38
BVO_CMOH-2m	3.5 ± 0.8	6.0	86.0	1.68	0.66	81.7	63.6	7.64
BVO_CMOH-4m	4.1 ± 0.7	5.3	75.3	1.54	0.66	78.0	60.9	9.46
BVO_CMOH-8m	7.4 ± 1.9	5.0	70.4	1.48	0.66	74.6	60.9	9.12
BVO_CMOH-16m	9.2 ± 2.1	4.3	60.2	1.10	0.76	60.6	63.5	8.71

Figure S3 presents the XRD patterns of these BiVO_4_ samples. All of the samples exhibit similar XRD patterns, indicating the presence of monoclinic-phase BiVO_4_. No additional XRD peaks appeared indicating that the coated layer either possesses an amorphous structure, according to the literature, or its contribution to XRD signal is lower than the detection limit. Figure 1b and 1c shows the HR-TEM images of Bare BVO and BVO_CMOH-16m, and the good lattice fringes of 0.31 nm were observed for both samples, which can be identified as the (121) plane of monoclinic-phase BiVO_4_. By the comparison between the two samples, the coated layer on the surface of BVO_CMOH-16m can be identified as an amorphous structure, agreeing well with the previous reports.^18^ The TEM-EDS elemental mapping in Figure 1d and spectrum in Figure S4 of BVO_CMOH-16m demonstrated a uniform distribution of Co and Mn elements around the BiVO_4_ surface, revealing a homogeneous coating of CMOH layer on the BiVO_4_ electrode.

Figure S5a shows the UV–visible absorption spectra of Bare BVO and BVO samples with the ARD treatment. All samples exhibit nearly identical absorption profiles in the 400–550 nm range, corresponding to the band-to-band electronic transitions of BiVO_4_. Upon ARD treatment, increased absorption intensity at 400–550 nm and additional absorption tails extending from 550 to 650 nm are observed, which can be attributed to light absorption by the CMOH layer. Notably, the BVO_CMOH-16m sample displays pronounced absorption in this region, consistent with the presence of a relatively thick CMOH coating. The inset photographs further confirm that post-ARD treatment up to 8 min does not induce an obvious color change in the BiVO_4_ electrodes, whereas the BVO_CMOH-16m sample exhibits a distinct dark-yellow appearance. Tauc plot analysis (Figure S5b) estimates the optical bandgaps of all samples to be approximately 2.53 eV, indicating the absence of any significant spectral shift. These results suggest that the post-ARD treatment enhances visible-light absorption through the CMOH layer, while leaving the intrinsic electronic properties of the BiVO_4_ electrodes essentially unchanged. To further elucidate the nature of the CMOH–BiVO_4_ interface, the chemical states at the heterojunction were analyzed via XPS. Figure S6 shows the survey XPS spectra of Bare BVO and BVO_CMOH-16m. Clear Co and Mn signals emerge only in BVO_CMOH-16m, verifying successful CMOH deposition. Figure 2 presents high-resolution XPS spectra of Bare BVO and BVO_CMOH-16m. The Bi 4f spectra exhibit a slight shift toward lower binding energy upon CMOH deposition, suggesting interfacial electronic interactions between Bi and the CMOH layer. However, the V 2p XPS spectra between the Bare BVO and BVO_CMOH-16m show negligible shifts in binding energy, indicating the coordinated environment of vanadium remains unchanged. Note that the Raman spectra (Figure S7) of the Bare BVO and BVO_CMOH-16m were measured to confirm that both samples exhibit similar vibrational bands of VO_4_ tetrahedron without any variation to their crystalline structure. The O 1s spectrum of Bare BVO reveals three components: lattice oxygen (O_L_) at 529.3 eV, oxygen vacancies (O_V_) at 530.2 eV, and surface hydroxyl/water species (O_C_) at 531.7 eV. In BVO_CMOH-16m, the O_L_ peak remains at 529.3 eV, while the other components shift to 530.6 and 531.8 eV, attributed to O^2–^ and OH^–^ species from the CMOH overlayer.^29^ Consistent Co and Mn signals are evident only in the modified sample, confirming the compositional presence of the CMOH layer. The Mn 2p spectrum of BVO_CMOH-16m exhibits peaks at 642.0 and 653.5 eV, characteristic of Mn­(IV) species.^30^ The three peaks at 795.4, 780.1, and 789.8 eV in the Co 2p XPS spectrum of BVO_CMOH-16m are attributed to Co 2p_1/2_, Co 2p_3/2_, and Co 2p_3/2_ satellite of Co­(III) species, respectively.^31^ These results reveal that the CMOH present on the BiVO_4_ surface via the chemical bonding between oxygen species and Bi, which can also passivate the O_V_ of BiVO_4_. Therefore, this chemical bonding at the interface of BiVO_4_/CMOH may facilitate interfacial charge transfer behaviors and enhance charge separation of the BiVO_4_ photoanode in the PEC application.

**Figure 2:**
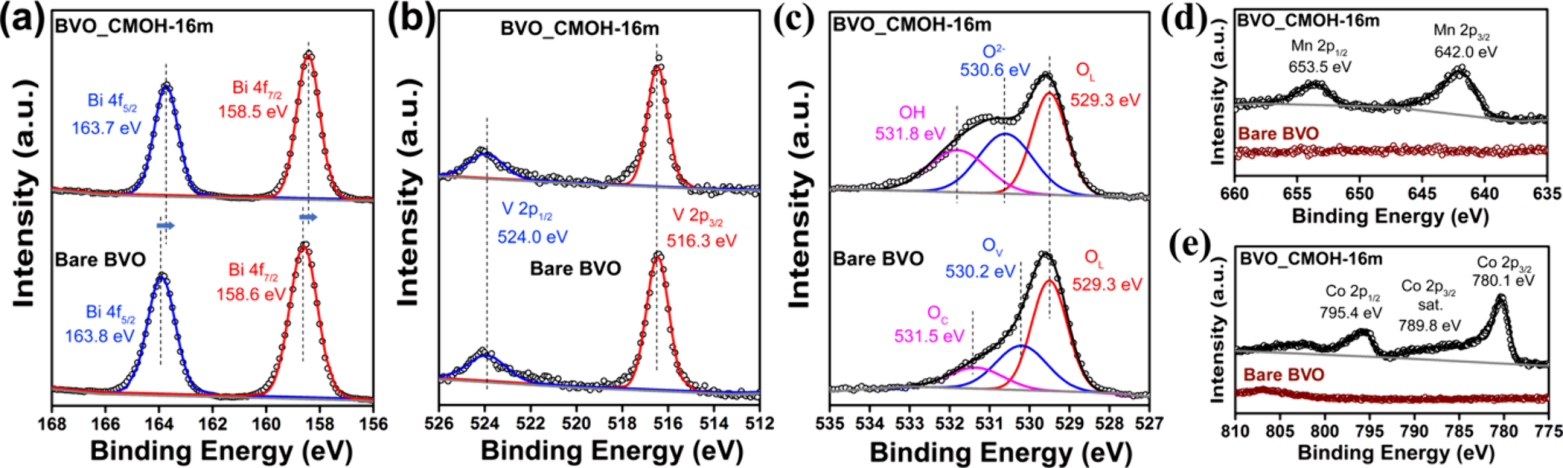
(a) Bi 4f, (b) V 2p, (c) O 1s, (d) Mn 2p and (e) Co 2p XPS spectra of Bare BVO and BVO_CMOH-16m.

The PEC examination was performed by using the Bare BVO and CMOH-coated BiVO_4_ samples as the working electrodes coupled with Pt and Ag/AgCl as the counter and reference electrodes, respectively. Figure 3a shows the linear sweep voltammetry (LSV) curves of the Bare BVO and CMOH-coated BiVO_4_ samples under dark and light illumination. Significantly, the onset potentials of CMOH-coated BiVO_4_ samples reduced to 0.22 V_RHE_ as compared to that of Bare BVO at 0.58 V_RHE_ under light illumination. The CMOH-coated BiVO_4_ electrodes showed a significant photocurrent density compared to Bare BVO in the same range of applied potential. The photocurrent density at 1.23 V_RHE_ for all of the test electrodes is summarized in Table 1. The optimal sample, BVO_CMOH-2m, presents an ∼2.6 times enhancement in photocurrent density as compared to Bare BVO at 1.23 V_RHE_. The incident-photo-to-current conversion efficiency (IPCE) spectra of the tested electrodes were collected under 1.23 V_RHE_ and are shown in Figure 3b. All the samples exhibit similar profiles within the range of 300–600 nm in the IPCE spectra. This phenomenon indicates that charge collection primarily occurs from the conduction band (CB) and valence band (VB) of BiVO_4_. The IPCE at 450 nm under 1.23 V_RHE_ for the test electrodes is also listed in Table 1. A 3.1-fold improvement in efficiency was observed when comparing the performance of Bare-BVO and BVO_CMOH-2m, which indicates that the photoactivity improvement of BiVO_4_ photoanodes can be attributed to the better photon-to-electron conversion by surface CMOH decoration. It is worth noting that no efficiency enhancement is observed in the 520–600 nm region for the CMOH-coated BVO electrodes. This result indicates that the increased absorbance of the CMOH layer in the 500–600 nm range does not contribute directly to the photocurrent. Instead, the enhanced photocurrent predominantly originates from improved photoactivity of the BiVO_4_ photoanode in the 300–500 nm spectral region following CMOH decoration.

**Figure 3:**
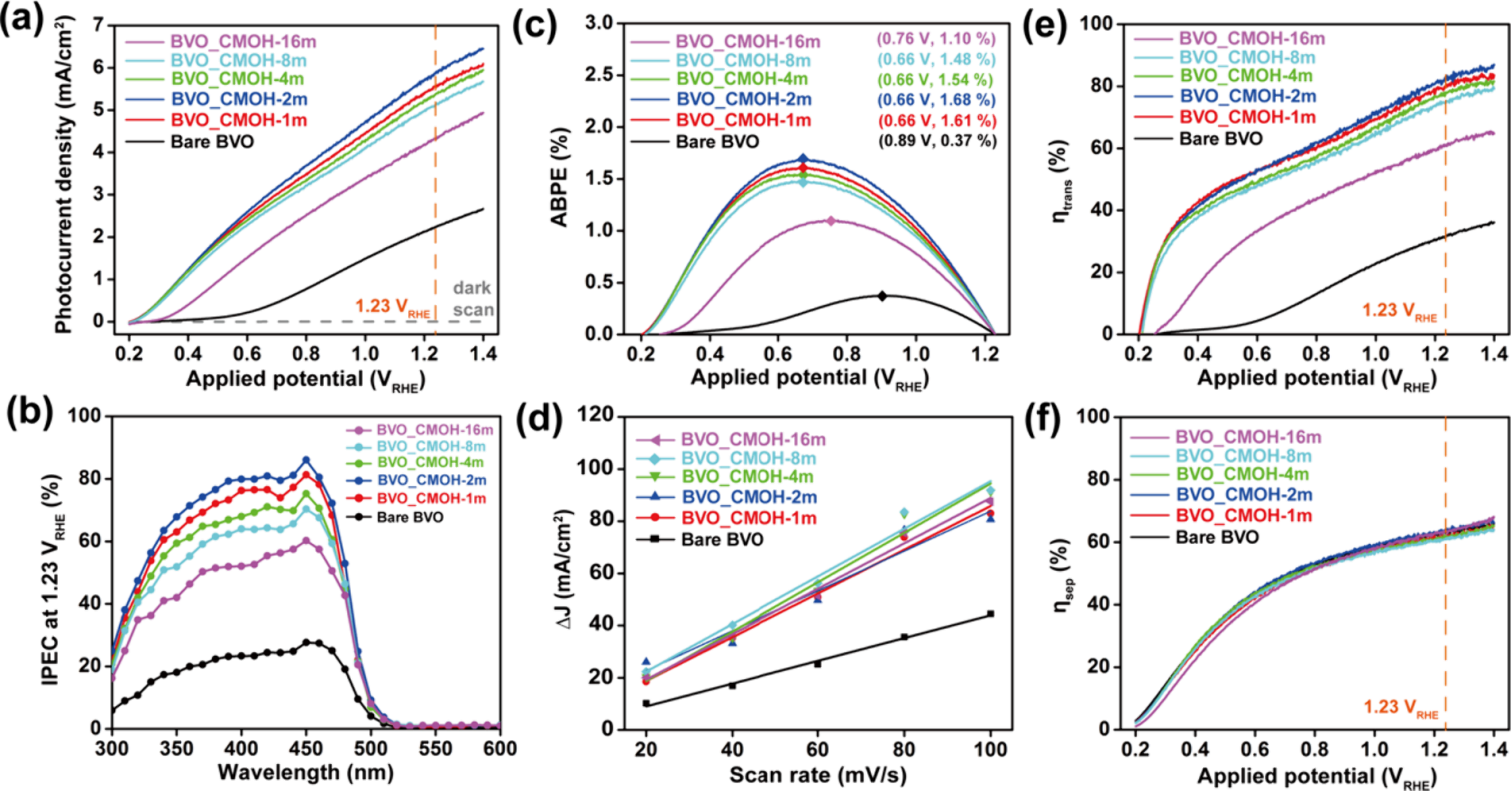
(a) LSV, (b) IPCE at 1.23 V_RHE_, (c) ABPE, (d) ESCA, (e) transfer efficiency, and (f) separation efficiency of the Bare BVO, BVO_CMOH-1m, BVO_CMOH-2m, BVO_CMOH-4m, BVO_CMOH-8m and BVO_CMOH-16m in PEC water oxidation.

The applied bias photon-to-current efficiency (ABPE) of the Bare BVO and CMOH-coated BiVO_4_ samples was calculated and is shown in Figure 3c. The highest ABPE and the corresponding applied voltage for all of the test electrodes are summarized in Table 1. An ∼4.5-fold enhancement was observed when comparing ABPE between BVO_CMOH-2m and Bare BVO. These results indicate that the photoactivity and efficiency of the BiVO_4_ electrode are dramatically improved after CMOH coating on its surface. To understand the argument regarding surface area effects on photoactivity enhancement, the electrochemical surface area (ECSA) of Bare BVO and CMOH-coated BiVO_4_ samples was measured and is shown in Figure 3d. The double-layer capacitance (C_dl_) of Bare BVO and CMOH-coated BiVO_4_ samples was calculated and is shown in Table 1. Apparently, the C_dl_ of CMOH coated BVO samples was about 2-times higher than that of Bare BVO. Therefore, the CMOH layer would improve the ion adsorption of the BiVO_4_ photoanodes, leading to better photoactivity in the PEC water oxidation. Furthermore, the effects of CMOH layer deposition on the photogenerated charge carrier of the BiVO_4_ photoanode were investigated by measuring the charge transfer (η_trans_) and separation (η_sep_) efficiencies for Bare BVO and CMOH-coated BiVO_4_ samples in an electrolyte containing Na_2_SO_3_ as a sacrificial hole scavenger (LSV curves are shown in Figure S8). As shown in Figure 3e and 3f, the CMOH-coated BiVO_4_ electrodes present a better η_trans_ than the Bare BVO electrode under the same range of applied potential. The η_trans_ of the optimal sample, BVO_CMOH-2m, presents an ∼2.6-fold improvement as compared to that of Bare BVO under the same applied potential. However, all the test electrodes show similar η_sep_ under the same condition. The values of η_trans_ and η_sep_ at 1.23 V_RHE_ are listed in Table 1. This result further confirms that the CMOH layer coating would not affect the internal charge generation behavior while significantly improving hole transfer from BiVO_4_ into electrolyte for carrying out OER.

Since the CMOH coating is expected to influence the charge-carrier behavior of BiVO_4_ photoanodes during PEC operation, the associated charge-carrier dynamics were investigated using IMPS.^32,33^ IMPS spectra of Bare BVO and the optimized BVO_CMOH-2m sample were collected over an applied potential range of 0.2–0.8 V_RHE_ to probe their interfacial charge-transfer characteristics, as shown in Figures 4a and 4b. For Bare BVO, the IMPS response exhibits a nonmonotonic real component, Re­{H}, at 0.2 and 0.4 V_RHE_, whereas a monotonic Re­{H} behavior is observed at higher potentials of 0.6 and 0.8 V_RHE_. This behavior can be attributed to the insufficient driving force of photogenerated holes to overcome the overpotential required for water oxidation when the applied bias is below the onset potential. In contrast, the Re­{H} response of BVO_CMOH-2m shows a monotonic increase across the entire potential range, indicating that the introduction of the CMOH layer effectively reduces the interfacial overpotential and promotes hole-transfer kinetics at the BiVO_4_/electrolyte interface.^34^

**Figure 4:**
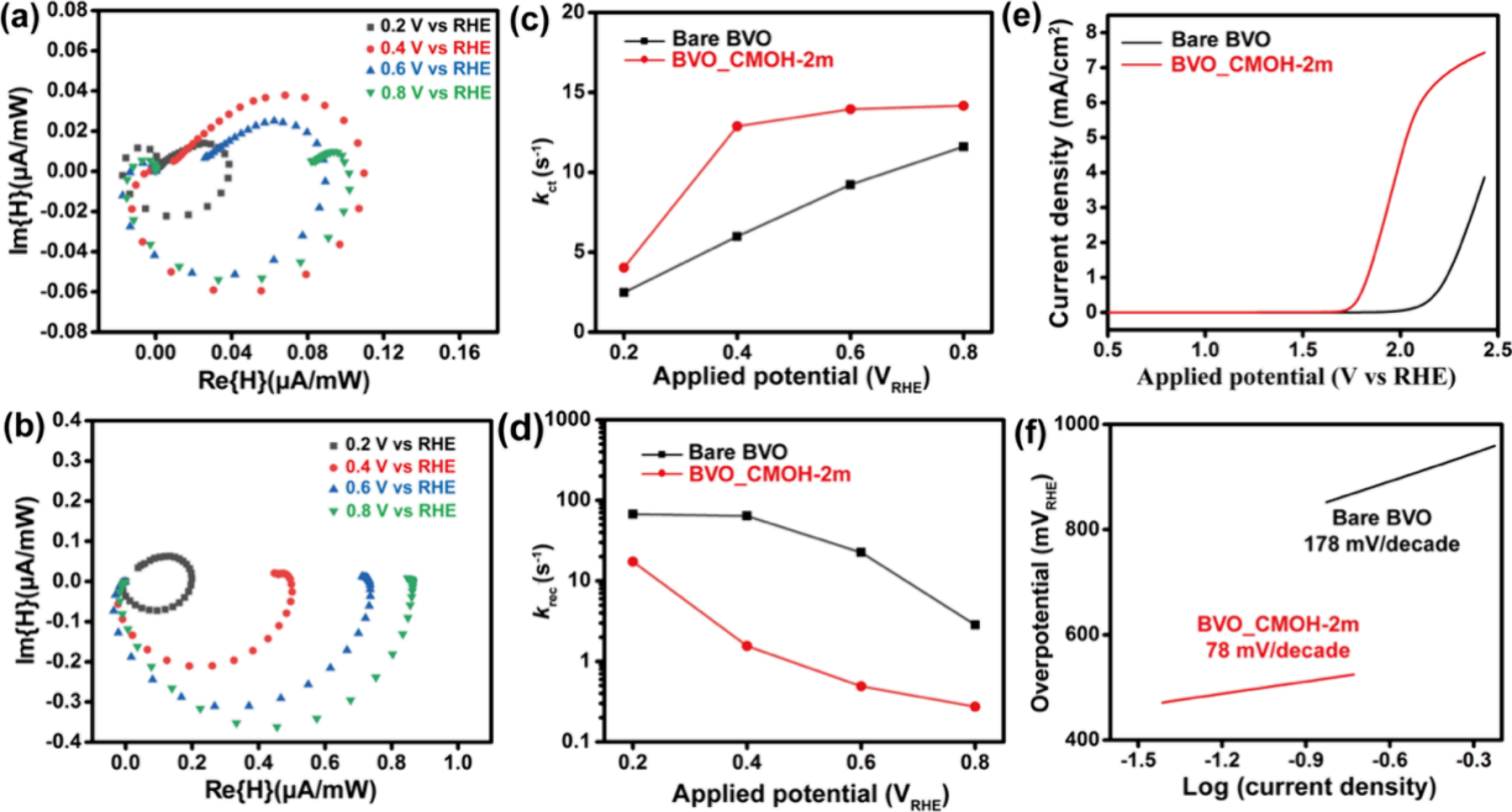
IMPS spectra of (a) Bare BVO and (b) BVO_CMOH-2m at various potentials. (c) Rate constant for charge transfer (*k*_ct_) and (d) rate constant for charge recombination (*k*_rec_) of Bare BVO and BVO_CMOH-2m. (e) Polarization Curve and (f) Tafel plots of Bare BVO and BVO_CMOH-2m.

To study the interfacial charge carrier dynamics, the obtained IMPS spectra can be further calculated, resulting in the charge transfer rate constant (*k*_ct_) and surface recombination rate constant (*k*_rec_) of the Bare BVO and BVO_CMOH-2m electrodes in PEC water oxidation, as shown in Figure 4c and 4d. These calculated values are also summarized in Table 2. The slow *k*_ct_ and fast *k*_rec_ of Bare BVO reveal that significant charge recombination via surface states occurred, suppressing the interfacial charge transfer. As the applied bias increased for Bare BVO, the charge recombination would be inhibited, and charge transfer at the interface would be promoted. On the other hand, BVO_CMOH-2m shows higher *k*_ct_ values and over one order decreased *k*_rec_ as compared to that of Bare BVO. This outcome reveals that the CMOH layer may passivate the surface states of BiVO_4_, resulting in a promoted charge separation and transfer at the interface between BVO_CMOH-2m and electrolyte.^33^ Furthermore, the dark scan of Bare BVO and BVO_CMOH-2m was collected. As shown in Figure 4e, the significantly reduced on-set potential for BVO_CMOH-2m at 1.79 V_RHE_ compared to Bare BVO at 2.20 V_RHE_ under dark conditions was measured (estimation from tangent method). The Tafel slope (Figure 4f) was further calculated as 78 and 178 mV/dec for BVO_CMOH-2m and Bare BVO, respectively. The reduced Tafel slope suggests that the CMOH layer promotes interfacial charge transfer at the BVO_CMOH-2m/electrolyte interface, thereby enhancing the water oxidation kinetics and improving the photoactivity of the CMOH-coated BiVO_4_ photoanode. The performance of BVO_CMOH-2m was further benchmarked against those of previously reported advanced BiVO_4_ photoanodes in PEC OER.^35–38^

**Table 2:** Calculation Results of Charge Carrier Kinetics of Bare BVO and BVO_CMOH-2m Based on the IMPS Measurements

	Applied Potential (V_RHE_)							
	Bare BVO				BVO_CMOH-2m			
	0.2	0.4	0.6	0.8	0.2	0.4	0.6	0.8
*k*_ct_ (s^–1^)	2.5	6.0	9.2	11.6	4.0	12.9	13.9	14.2
*k*_rec_ (s^–1^)	67.5	63.9	22.5	2.8	17.4	1.6	0.5	0.3

Based on the PEC performance, IMPS, and Tafel analyses, a plausible mechanism underlying the enhanced PEC water oxidation activity of the CMOH-deposited BiVO_4_ photoanode is proposed, as illustrated in Figure 5. For the Bare BVO, illumination generates electron–hole pairs in the CB and VB, respectively. A substantial fraction of these photogenerated charge carriers is rapidly trapped (within picoseconds) by surface defect states, including shallow electron traps associated with oxygen vacancies (O_V_) and hole traps related to VO^2+^/VO^+^ species, which are collectively referred to as recombination surface states (r-SS).^39^ Under a low applied bias of 0.6 V_RHE_, the interfacial electric field is insufficient to overcome the kinetic overpotential for OER, resulting in severe surface recombination through these trap states and a high *k*_rec_ value of 22.5 s^–1^. Only a small fraction of charge carriers participates in interfacial reactions, where CB electrons migrate to the Pt counter electrode to drive H_2_ evolution, while VB holes oxidize water in the electrolyte, yielding a relatively low *k*_ct_ of 9.2 s^–1^. Consequently, the Bare BVO photoanode exhibits a low ABPE of 0.14% at 0.6 V_RHE_. Even at an optimal applied potential of 0.89 V_RHE_, the pronounced charge recombination and inefficient hole transfer limit the ABPE to 0.37%.

**Figure 5:**
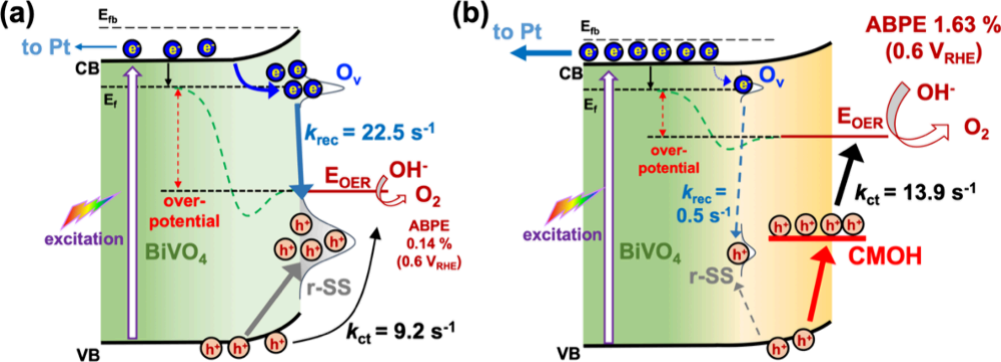
Schematic illustration for the proposed mechanism of charge carrier dynamics at the interfaces of (a) Bare BVO/electrolyte and (b) BVO_CMOH-2m/electrolyte in PEC OER at an applied voltage of 0.6 V_RHE_.

In contrast, for the BVO_CMOH-2m electrode, the CMOH OEC overlayer effectively passivates surface trap states, substantially enhancing charge separation and suppressing interfacial recombination, as evidenced by a dramatically reduced *k*_rec_ of 0.5 s^–1^. The photogenerated electrons in the CB of BiVO_4_ readily migrate to the Pt counter electrode for H_2_ evolution, while the accumulated holes in the VB are efficiently extracted through the CMOH OEC layers and subsequently injected into the electrolyte. The Co^3+^ species within the CMOH layer are proposed to act as hole-transport mediators, facilitating interfacial hole transfer with a *k*_ct_ of 13.9 s^–1^ and thereby promoting efficient water oxidation. In addition, the CMOH interlayer is expected to modulate the interfacial Fermi level, further reducing the overpotential required for the oxygen evolution reaction. As a result, the BVO_CMOH-2m photoanode achieves a remarkable ABPE of 1.63% at a low applied potential of 0.6 V_RHE_, representing more than an order-of-magnitude improvement compared to Bare BVO. Overall, the synergistic effects of reduced overpotential, suppressed charge-carrier recombination, and enhanced hole-transfer kinetics account for the superior PEC OER performance (ABPE 1.68%) of the BVO_CMOH-2m photoanode at a low applied potential (0.66 V_RHE_).

To demonstrate the general applicability of the CMOH layer for enhancing photoanodes active in PEC water oxidation, additional metal oxide photoanodes, including WO_3_ and ZnO, were examined. Figure S9 presents the LSV curves of these metal oxides with and without the CMOH coating under illumination. Both WO_3_ and ZnO photoanodes exhibit noticeably enhanced photoactivity after CMOH decoration, indicating that this facile ARD strategy can effectively improve the PEC performance of a broad range of typical metal oxide photoanodes. This should be mainly due to (1) the high optical transparency coming from the ultrathin thickness of CMOH coating (3.5 nm), (2) the general strong binding capability of CMOH to metal oxide surfaces, and (3) the intrinsically enhanced hole-transfer kinetics of metal oxide photoanodes by CMOH.

In addition to activity enhancement, the operational stability of the CMOH-coated BiVO_4_ photoanode was evaluated to assess its potential for practical PEC applications. Figure S10 shows the chronoamperometric current–time (I–t) responses of Bare BVO and BVO_CMOH-2m during 1 h of continuous operation at their respective optimal applied potentials corresponding to the highest ABPE values. For a clearer comparison, the photocurrent is normalized and plotted as the decay ratio versus illumination time, as shown in Figure S11. The Bare BVO photoanode exhibits a pronounced photocurrent decay of approximately 70%, which is attributed to the well-known vanadium leaking-induced photocorrosion of BVO in the absence of effective cocatalyst protection.^40^ The XRD, XPS, SEM and HR-TEM characterizations were further performed to confirm the photocorrosion of the Bare BVO photoanode after 1 h of operation (Figure S12). Notably, we intentionally employed a literature-standard, broadly reproducible BiVO_4_ preparation (two-step fabrication process)^41,42^ without specialized defect/passivation engineering to benchmark interfacial stabilization under conditions representative of “typical-lab” photoanodes. Accordingly, the relatively rapid decay of Bare BVO serves as a stringent stress test that amplifies the sensitivity to interface design and allows the stability gains induced by the CMOH overlayer to be assessed in a manner that is both practical and generalizable. Although some research works have reported specialized approaches to inhibit this photocorrosion, many of these methods are usually complicated or only work for a specific material/system. In contrast, the BVO_CMOH-2m electrode shows a significantly reduced photocurrent decay of ∼40%. Notably, within the first 5 min of operation, the photocurrent decay of BVO_CMOH-2m is limited to ∼15%, substantially lower than that of Bare BVO (∼50% decay). These results indicate that the CMOH overlayer not only promotes interfacial charge transfer but also provides catalytically active sites for water oxidation, thereby mitigating photocorrosion of the BiVO_4_ photoanode. Similarly, XRD, XPS, SEM, and HR-TEM characterizations were also performed for BVO_CMOH-2m after 1 h of operation, and the results are shown in Figure S13. No significant photocorrosion of the BiVO_4_ photoanode was observed, although slight consumption and reconstruction of the CMOH layer was detected. This outcome may be attributed to the involvement of the OH^–^ species in the CMOH OEC during the OER process. Further studies may need to focus on the chemical stability improvement of the CMOH-coated BiVO_4_ photoanode during PEC OER. Based on the earlier results, one may also expect that such antiphotocorrosion behavior is also possible for WO_3_, ZnO and other metal oxide photoanodes.

This ARD-derived CMOH system offers a unique platform to interrogate photoelectrochemical interfacial phenomena that are inaccessible to conventional electrocatalysis. The amorphous structure, mixed-valence redox chemistry, and conformal interfacial contact of CMOH enable continuous electronic coupling with the typical two-step fabricated BiVO_4_,^43^ allowing the catalyst layer to function not only as an OEC but also as an active mediator of photogenerated hole extraction. The optimal ARD dip-coating process results in the state-of-the-art BiVO_4_ photoanode with CMOH OECs in PEC water oxidation (see Table S1). Although the stability of BiVO_4_ is markedly improved by CMOH modification, further enhancement of the long-term durability is still required. This may be achieved by incorporating additional transition metals, such as Fe and Ni, into the CMOH layer using the ARD strategy. Our previous work has demonstrated that Ag and Ce can be successfully incorporated with Co and Mn via ARD to fabricate highly active electrocatalysts for oxygen evolution.^20^ Accordingly, future efforts will focus on optimizing the metal composition deposited on the BVO to achieve superior chemical stability under PEC operation. Moreover, a systematic investigation of the individual roles of each metal ion is necessary to elucidate the mechanistic origins of the observed improvements in photoactivity and stability.

In summary, we demonstrate that an ultrathin amorphous CMOH layer deposited by ARD can fundamentally regulate interfacial charge-carrier dynamics at BiVO_4_ photoanodes. The redox flexibility of CMOH suppresses surface hole accumulation, passivates recombination-active surface states, and accelerates interfacial hole transfer—an effect distinct from that of conventional electrocatalytic behavior. A conformal ∼3.5 nm CMOH overlayer is rapidly formed within 2 min under ambient conditions without altering the structural or optoelectronic properties of BiVO_4_, delivering a photocurrent density of ∼6.0 mA cm^–2^ at 1.23 V_RHE_ and an ABPE of 1.68% at 0.66 V_RHE_. More broadly, this work establishes ARD as a universal, scalable strategy for integrating amorphous OEC with metal oxide photoanodes, providing a practical pathway toward large-area photoelectrochemical water splitting.

## Supplementary Material



## Notes

Experimental methods, SEM and TEM images of Bare BVO, histograms of thickness distribution of the coated CMOH layer, XRD patterns, UV–vis absorption spectra and Tauc plots for Bare BVO, BVO_CMOH-1m, BVO_CMOH-2m, BVO_CMOH-4m, BVO_CMOH-8m and BVO_CMOH-16m, EDS spectrum of BVO_CMOH-16m, Survey XPS, Raman spectra and chronoamperometric current–time (I–t) of Bare BVO and BVO_CMOH-16m, XRD patterns, XPS spectra, SEM and HR-TEM characterization of Bare BVO and BVO_CMOH-2m before and after 1 h PEC OER operation, and table of PEC performance comparison of BiVO_4_ photoanodes with various OEC-decoration (PDF)
