# Supplementary material for: Universal Interfacial Engineering via Amorphous Inorganic Binders: Passivating Surface States and Accelerating Hole Transfer across Metal Oxide Photoanodes in Photoelectrochemical Water Oxidation
Source: J Phys Chem Lett. 2026 Apr 17;17(17):5139–47. doi: 10.1021/acs.jpclett.6c00767 (PMC13137234; doi:10.1021/acs.jpclett.6c00767)
Supplement: Supplementary file 1 [file jz6c00767_si_001.pdf]

Supporting Information

Universal Interfacial Engineering via Amorphous Inorganic Binders: Passivating Surface States and Accelerating Hole Transfer across Metal Oxide Photoanodes in Photoelectrochemical Water Oxidation

Po-Keng Hsiao[a]‡, Yen-Lun Kung[a]‡, Yun-Pei Liu[b]‡, Shih-Wen Tseng[c], Tetsu Yonezawa[d,e], Chun-Hu Chen[b,f]* and Ying-Chih Pu[a,f]*

[a] Department of Materials Science, National University of Tainan, Tainan 700301, Taiwan

[b] Department of Chemistry, National Sun Yat-sen University, Kaohsiung 80424, Taiwan

[c] Core Facility Center of National Cheng Kung University, Tainan 70101, Taiwan

[d] Division of Materials Science and Engineering, Faculty of Engineering, Hokkaido University, Hokkaido 060-8628, Japan

[e] Department of Chemical Engineering, Faculty of Engineering, Chulalongkorn University, Phayathai Road, Pathumwan, Bangkok 10330, Thailand

[f] Green Hydrogen Research Center National Sun Yat-sen University Kaohsiung 80424, Taiwan

Experimental methods

Preparation of $BiVO_4$ photoanodes (Bare BVO)

The two-step process was used to prepare the $BiVO_4$ photoanodes that has been reported in our pervious study.[1] For the first step, the BiOI was coated on fluorine-doped tin oxide (FTO) substrate by electrodeposition. The dark brown plating solution was prepared by mixing two mixtures. One is the 5 mL aqueous solution that contained 0.45M of $Bi(NO_3)_3$ and 0.8 M KI (The pH value of this mixed solution was adjusted to 1.7 by HNO_3 solution). The other mixture is the 0.23 M p-benzoquinone dissolved in 1.75 mL of absolute ethanol and 0.25 mL ethylene glycol. The electrodeposition was performed in a potentiostat (Metrohm, Autolab PGSTAT302N) by using FTO as working electrode, Ag/AgCl as reference electrodes and Pt as counter electrodes. The applied potential was − 0.25 V vs Ag/AgCl for 180 seconds to form BiOI on the FTO substrate. The obtained BiOI electrodes were rinsed with deionized water (DI-H_2O) and ethanol and then dried with N_2 gas for the further utilization. For the second step, 0.15 mL of 0.2 M vanadyl acetylacetonate solution (in dimethyl sulfoxide) was covered on the BiOI electrode by drop-casting method and dried on a hotplate (Temperature was set as 150°C). The as-prepared electrode was calcined at 450 °C for 2 hours under air atmosphere. After the samples cooled down to room temperature, the excess V_2O_5 on $BiVO_4$ electrode was removed by soaking it into a 1 M NaOH solution for 20 min and then dried by N_2 for further characterization and utilization. The resulted $BiVO_4$ electrode sample was denoted as Bare BVO.

Preparation of CMOH coated $BiVO_4$ photoanodes (BVO_CMOH-xm)

The CMOH film precursor solution was prepared following a procedure adapted from previous literature.[2] First, 1.49 g of cobalt(II) acetate hexahydrate was dissolved in 75 mL of DI-H_2O to form Solution A. 0.317 g of potassium permanganate was dissolved in another 75 mL of DI-H_2O to form Solution B. The Solution A and B were mixed under stirring at 500 rpm for 5 minutes at room temperature. The as-prepared Bare BVO was subsequently immersed in this mixed solution for different soaking durations of 1, 2, 4, 8 and 16 minutes for

CMOH deposition, respectively, under normal ambient conditions . After the soaking process, the samples were removed from the mixed solution and washed by DI-H_2O and then dried by N_2 for further characterization and utilization. The resulted samples were denoted as $BiVO_4$_CMOH-xm (x = 1, 2, 4, 8 and 16).

Material Characterization

Morphological characterization of the Bare BVO and BVO_CMOH-xm samples was conducted using a high-resolution field-emission scanning electron microscope (FE-SEM, HITACHI SU-5000). High-resolution transmission electron microscopy (HR-TEM), together with energy-dispersive X-ray spectroscopy (EDX), was employed to examine the lattice spacing and elemental distribution of the Bare BVO and BVO_CMOH-xm samples on a JEOL JEM-2100F TEM equipped with a field emission gun. The crystal structure of the Bare BVO and BVO_CMOH-xm samples was analyzed by X-ray diffraction (XRD) using a Rigaku Americas Miniflex Plus powder diffractometer operated at 40 kV and 30 mA. Data were collected over a 2θ range of 10–80° at a scanning rate of 2° min^{-1}. UV–visible absorption spectra of the Bare BVO and BVO_CMOH-xm samples was recorded using a micro-spectrometer (RLS1000, Rainbow-Light) integrated with an integrating sphere. X-ray photoelectron spectroscopy (XPS) measurements were performed on a Thermo Fisher Scientific ESCALAB Xi+ system with an Al Kα radiation source. Raman measurements were carried out on a Horiba Jobin Yvon IHR 550 to characterize the vibrational properties of the BiVO4 photoanodes with an excitation wavelength of 532 nm.

Photoelectrochemical (PEC) measurements

The uncoated FTO region of the $BiVO_4$ photoanodes was electrically connected to a conductive wire and sealed with insulating epoxy resin, yielding an exposed $BiVO_4$ active area of ~0.7 cm^2. The as-prepared electrodes were used as working electrodes for PEC measurements, with a Pt wire and an Ag/AgCl electrode serving as the counter and reference electrodes, respectively. A 0.5 M H_3BO_3 aqueous solution (pH 9.5) was employed as the electrolyte. For the evaluation of transfer efficiency (η_{trans}) and charge separation efficiency (η_{sep}), photocurrent measurements

were conducted in an electrolyte containing 0.5M of H_3BO_3 aqueous solution (pH 9.5) and 0.2 M of Na_2SO_3 as a sacrificial hole scavenger. Linear sweep voltammetry (LSV), chronoamperometry, electrochemical impedance spectroscopy (100 kHz to 0.1 Hz), and Mott–Schottky measurements (1 kHz) were conducted using a potentiostat (Metrohm, Autolab PGSTAT302N) under simulated AM 1.5G illumination (300 W Xe lamp (Excelitas HX22) equipped with AM 1.5 and infrared water filters). Intensity-modulated photocurrent spectroscopy was performed using the same potentiostat coupled with an LED driver kit (AUT.LED.LDC470) over a frequency range of 20 kHz to 30 mHz. Incident photon-to-electron conversion efficiency (IPCE) measurements were carried out using the same potentiostat coupled with a solar simulator (HORIBA PowerArc) and a monochromator (HORIBA TRIAX 180). Gas evolution experiments were conducted in a gas-tight PEC cell (custom-made by Gingen Technology) separated by a Nafion membrane to isolate the $BiVO_4$ photoanode and Pt cathode for O_2 and H_2 evolution, respectively. The evolved gases were collected using a gas-tight syringe and quantified by gas chromatography (Shimadzu GC-2010, BID-2010 Plus). The calculations of V_{RHE} potential, η_{trans}, η_{sep}, applied bias photo-to-current efficiency (ABPE), incident-photo-to-current conversion efficiency (IPCE), intensity-modulated photocurrent spectroscopy (IMPS) and Faradaic efficiency are shown as below:[3-5]

1. The conversion between potentials vs Ag/AgCl and those vs reversible hydrogen electrode (RHE) is calculated using the equation:

$$E_{RHE} = E_{Ag/AgCl} + E_{Ag/AgCl\ vs.\ SHE} + 0.059 \times pH$$

2. ABPE is performed by using the equation below:

$$ABPE(\%) = \frac{J\left(\frac{mA}{cm^2}\right) \times (1.23 - V_b(V))}{P_{total}\left(\frac{mA}{cm^2}\right)}$$

3.Mott-schottky 0~0.6 V vs. RHE, charge carrier concentration (N_d) can be calculated by using the equation below:

$$N_d = \frac{2}{q\varepsilon\varepsilon_0}\left(\frac{d\left(\frac{1}{C^2}\right)}{dV_s}\right)^{-1}$$

Where q is electric charge of 1.6×10^{-19} C, ε is dielectric constant of $BiVO_4$ as 68 F cm^{-2}, ε_0 is the Permittivity of free space of 8.854×10^{-14} F cm^{-1}, C is the capacitance of depletion layer，V_s is applied voltage, $\frac{d\left(\frac{1}{C^2}\right)}{dV_s}$ is the value of slope.

4. η_{trans} is calculated by the equation:

$$\eta_{\text{trans}}\ (\%) = \frac{J_{electrolyte}}{J_{scavenger}} \times 100$$

5. The formula for computing the η_{sep} is given by:

$$\eta_{\text{sep}}\ (\%) = \frac{J_{scavenger}}{J_{max} \times \eta_{\text{abs}}} \times 100$$

Where J_{max} refers to the theoretical limit of the photocurrent under AM 1.5G (100 mW/cm²) illumination at an applied bias of 1.23 V vs.RHE. (and η_{abs} is measured as 67% at 300-500 nm.)

6. The equation for calculating the measured IPCE is expressed as:

$$\text{IPCE}(\%) = \frac{J\left(\frac{\mu A}{cm^2}\right) \times 1240}{\lambda(nm) \times P_{wavelength}\left(\frac{\mu W}{cm^2}\right)}$$

7. Intensity-Modulated Photocurrent Spectroscopy (IMPS):

Module used[6]:

$$H = \frac{I_0[k_t + i\omega(\frac{C_{cell}}{C_{sc}})]}{(k_t + k_r + i\omega)(1 + i\omega\tau)}$$

In this work, a simplified form was used by neglecting the C_{cell}/C_{sc} term, which is typically much smaller than unity and has a negligible influence on the dominant IMPS arc. This simplification allows the rate constants k_{ct} and k_{rec} to be extracted using the standard two-rate IMPS model.

The computation of the measurement data is based on the following equation:

$$\omega_{max} = 2\pi f_{max} = k_{ct} + k_{rec}$$

$$\eta_{IMPS} = LF/HF$$

$$k_{ct} = 2\pi \cdot f_{max} \cdot LF/HF$$

$$k_{rec} = 2\pi \cdot f_{max} - k_{ct}$$

Where f_{max} is the frequency at the peak of the arc, ω_{max} is the angular frequency corresponding to the maximum of the arc in the first quadrant, HF is the photocurrent value at the high-frequency side of the arc while LF is the photocurrent value at the low-frequency side of the same arc, k_{ct} is the charge-transfer rate constant extracted from the arc, k_{rec} is the recombination rate constant associated with the same arc. η_{IMPS} is the IMPS charge-transfer efficiency, calculated as the ratio of the low-frequency photocurrent (LF) to the high-frequency photocurrent (HF).

8. The equation for calculating the Faradaic efficiency (%)：

$$\text{Faradaic efficiency (\%)} = \frac{z \times n \times F}{Q} \times 100\%$$

In this equation, z represents the number of electrons required for gas evolution, with z = 4 for O_2 evolution and z = 2 for H_2 evolution. F is the Faraday constant (96,485 C mol^{-1}), and n is the molar amount of O_2 or H_2

evolved during the PEC reaction. For faradaic efficiency (FE) evaluation, the amounts of evolved gases were measured after 1 h of PEC operation. The total charge (Q) passed through the system was calculated by integrating the current over time.

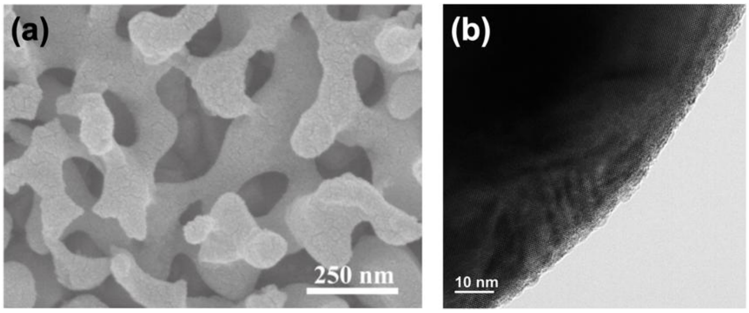

Figure S1. (a) SEM and (b) TEM images of Bare BVO.

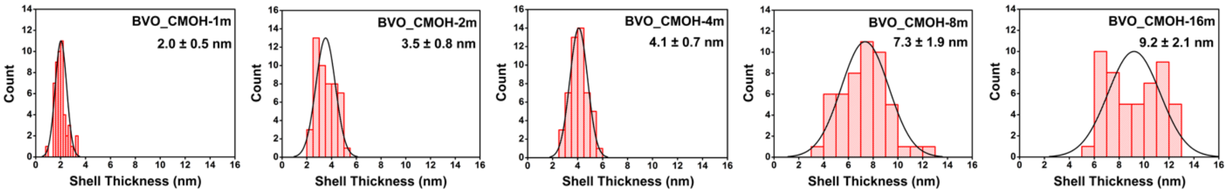

Figure S2. Histograms of thickness distribution of the coated CMOH layer on the surface of BVO_CMOH-1m, BVO_CMOH-2m, BVO_CMOH-4m, BVO_CMOH-8m and BVO_CMOH-16m, respectively.

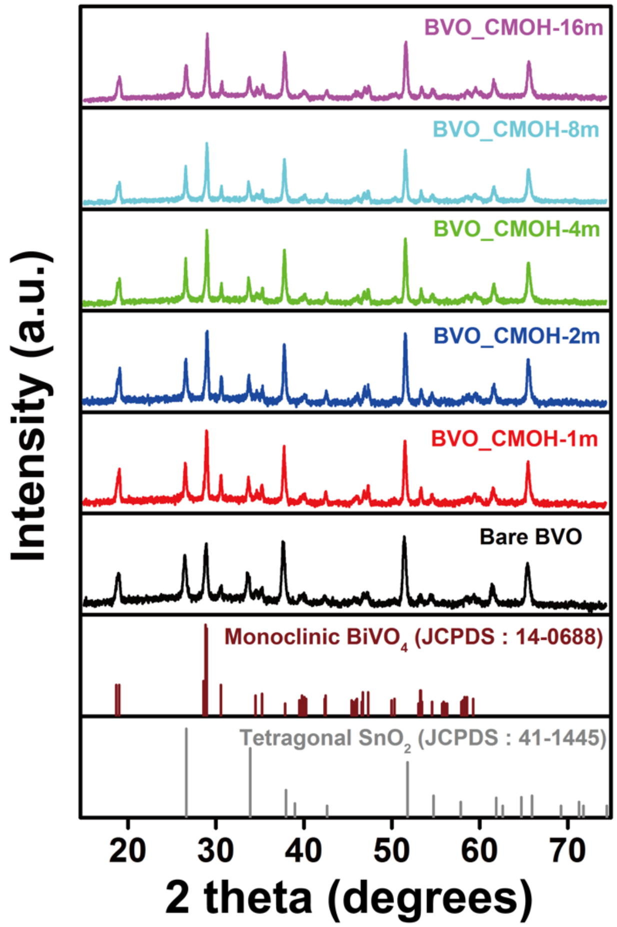

Figure S3. XRD patterns of Bare BVO, BVO_CMOH-1m, BVO_CMOH-2m, BVO_CMOH-4m, BVO_CMOH-8m and BVO_CMOH-16m (the standard patterns of monoclinic $BiVO_4$ and tetragonal SnO_2 (FTO) are incorporated for comparion).

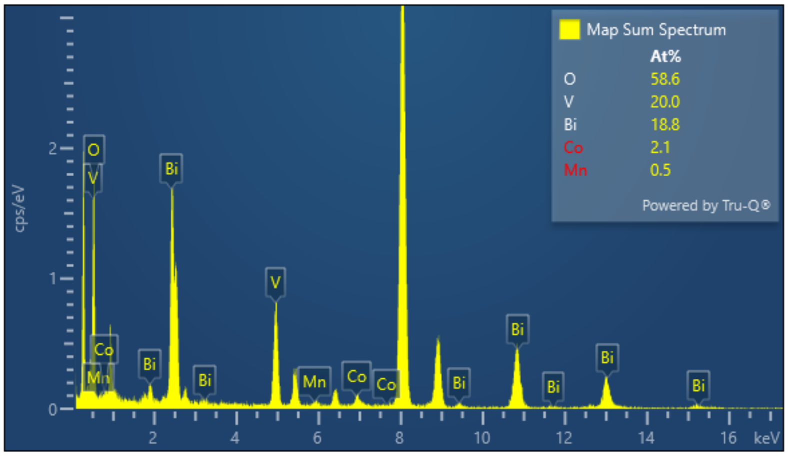

Figure S4. EDS spectrum of BVO_CMOH-16m

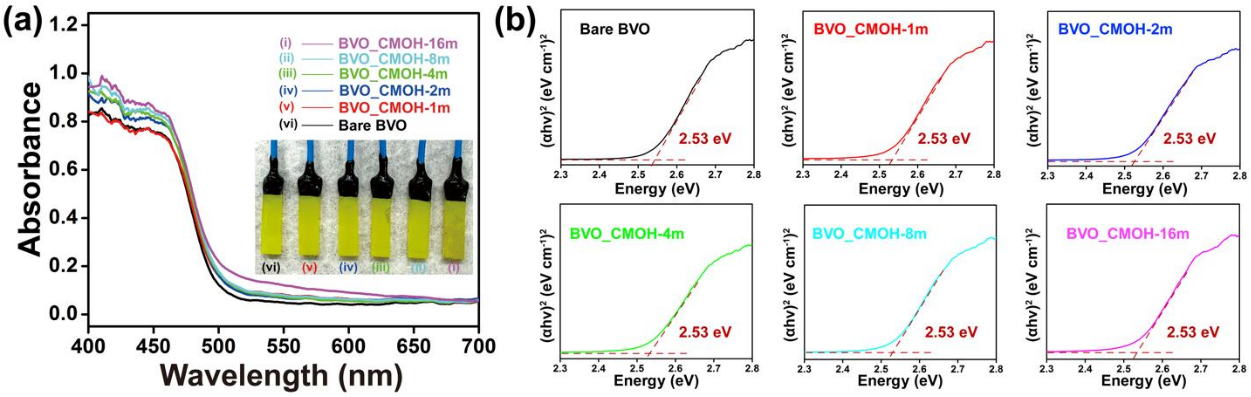

Figure S5. (a) UV–vis absorption spectra and (b) Tauc plots of Bare $BiVO_4$ BVO_CMOH-1m, BVO_CMOH-2m, BVO_CMOH-4m, BVO_CMOH-8m and BVO_CMOH-16m

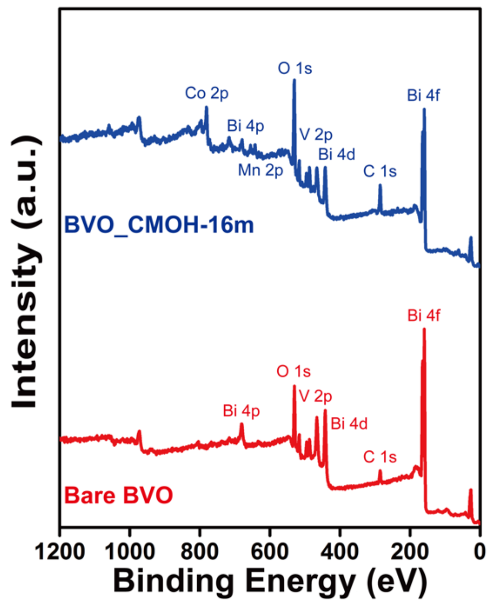

Figure S6. Survey XPS spectra of Bare BVO and BVO_CMOH-16m.

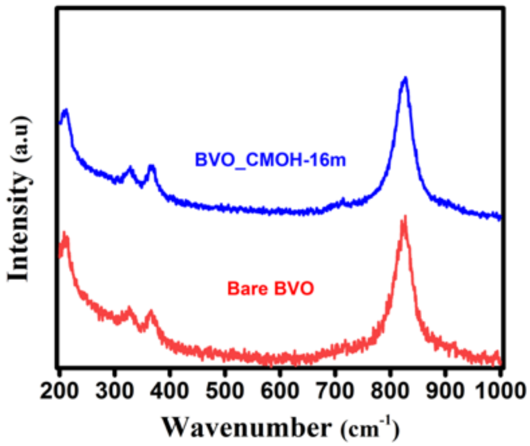

Figure S7. Raman spectra of Bare BVO and BVO_CMOH-16m.

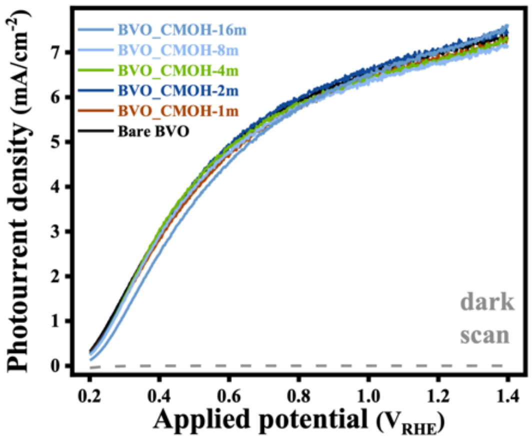

Figure S8. LSV of the Bare BVO, BVO_CMOH-1m, BVO_CMOH-2m, BVO_CMOH-4m, BVO_CMOH-8m and BVO_CMOH-16m in PEC water oxidation

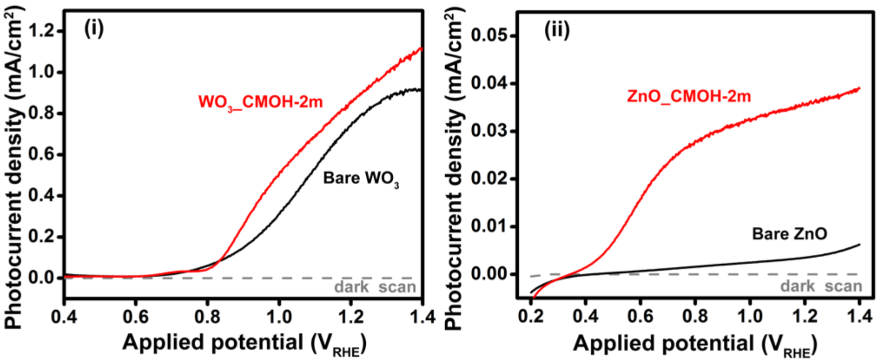

Figure S9. LSV of (i) WO_3 and (ii) ZnO photoelectrode with and without ARD treatment under illumination.

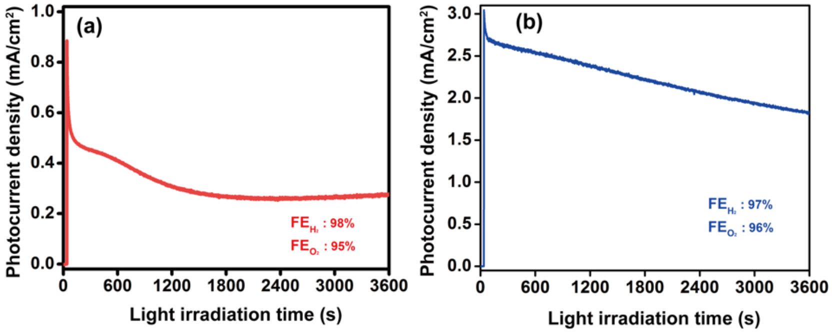

Figure S10. Chronoamperometric current–time (I–t) of the (a) Bare BVO (at 0.89 V_{RHE}) and (b) BVO_CMOH-2m (at 0.66 V_{RHE}). The insets are faraday efficiency of H_2 (FE_{H2}) and O_2 (FE_{O2}) evolution.

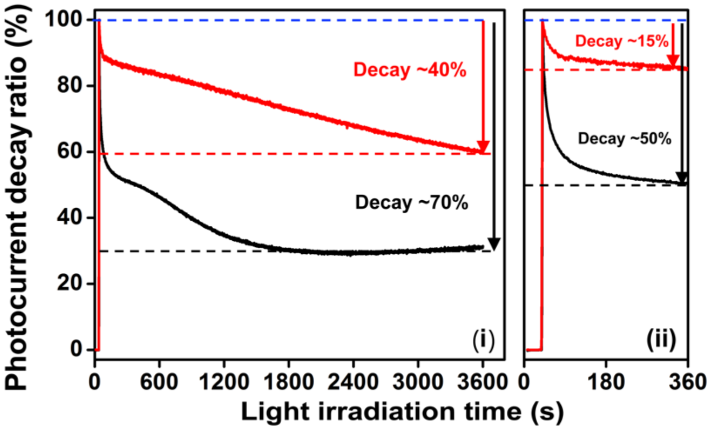

Figure S11. Normalized photocurrent decay ratio of the Bare BVO and BVO_CMOH-2m in chronoamperometric current–time (I–t) measurements.

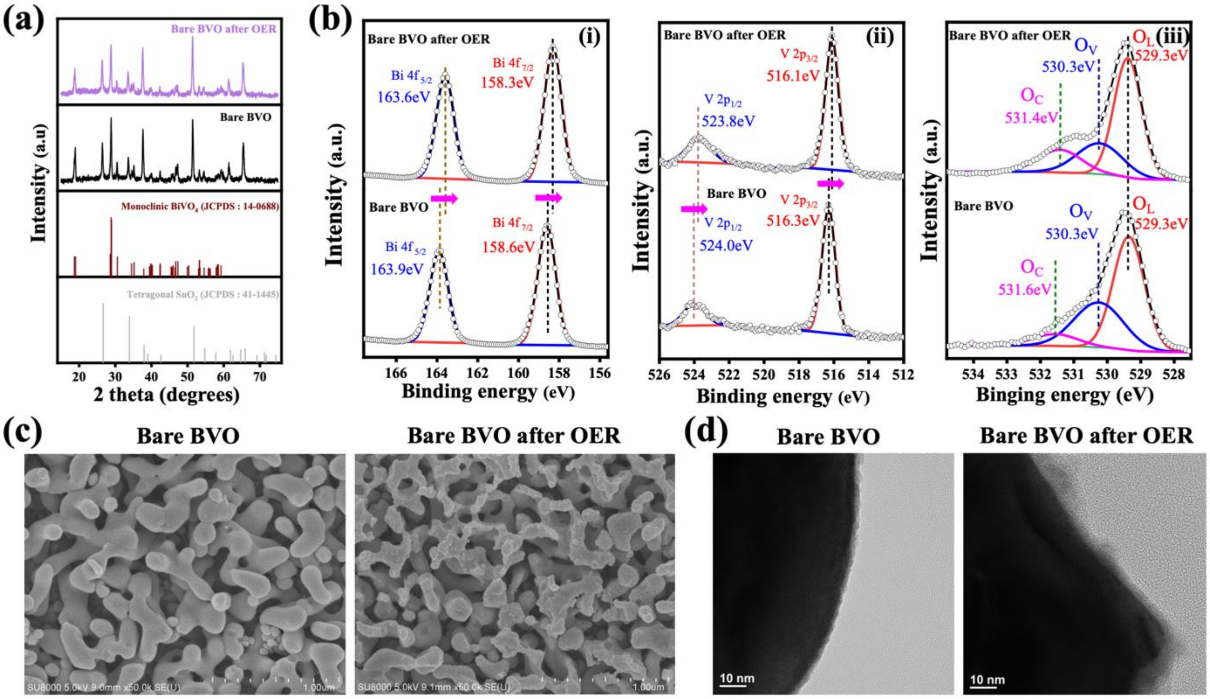

Figure S12. (a) XRD patterns, (b) XPS spectra of (i) Bi 4f, (ii) V 2p, and (iii) O 1s, (c) SEM images, and (d) HR-TEM images of Bare BVO before and after one hour of PEC OER operation under an applied potential of 0.89 V_{RHE}. After PEC OER operation, the crystal structure of monoclinic $BiVO_4$ showed no significant change. However, the Bi 4f and V 2p XPS spectra shifted toward lower binding energies. The O 1s XPS spectra exhibited a decreased contribution from the O_C peak (surface hydroxyl/water species) and an increased contribution from the O_V peak (oxygen vacancies). In the SEM images, the particle size of the top $BiVO_4$ layer became smaller after operation. In addition, an amorphous surface layer was observed on $BiVO_4$ in the HR-TEM images. These results indicate that photocorrosion occurred in Bare BVO due to vanadium leaching, leading to an increase in oxygen vacancies and surface reconstruction.

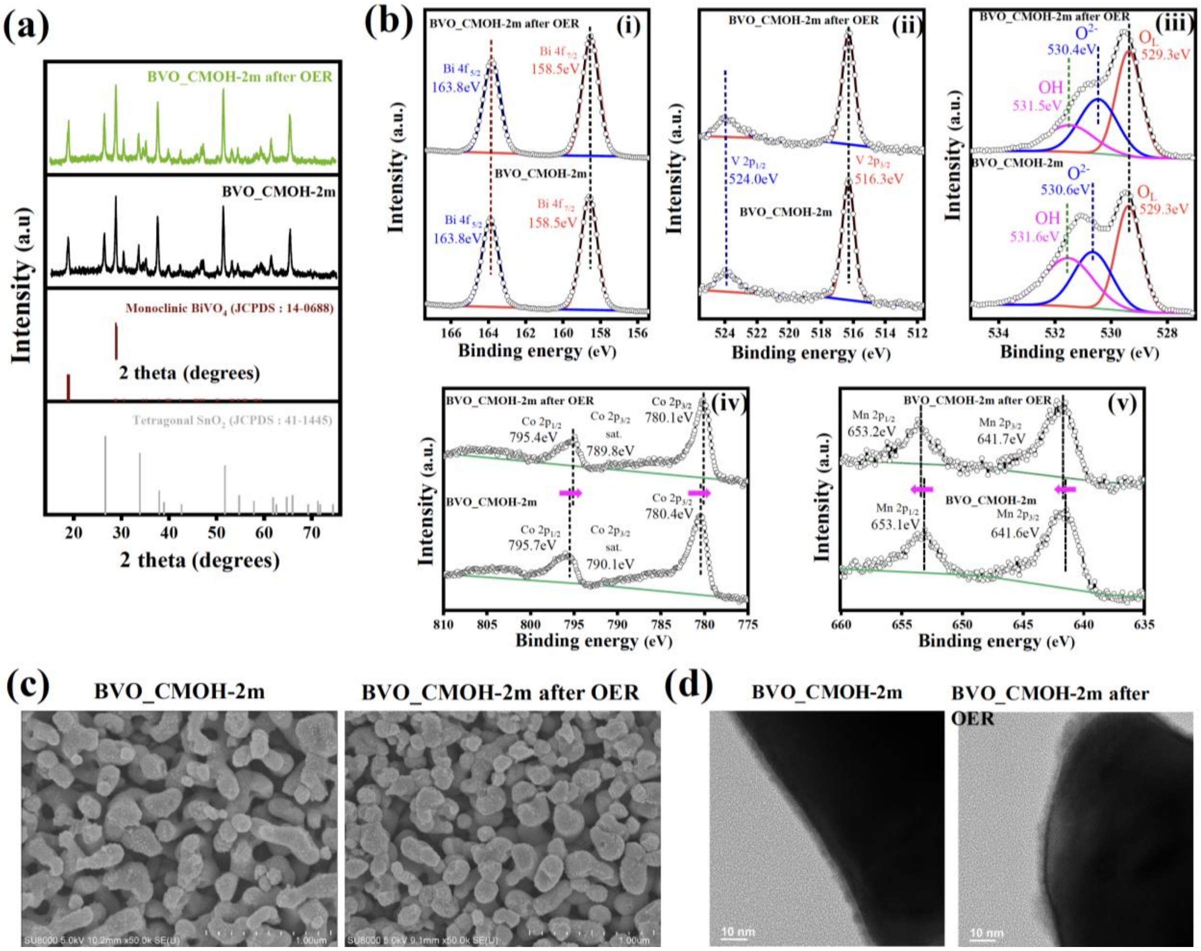

Figure S13. (a) XRD patterns, (b) XPS spectra of (i) Bi 4f, (ii) V 2p, (iii) O 1s, (iv) Co 2p, and (v) Mn 2p, (c) SEM images, and (d) HR-TEM images of BVO_CMOH-2m before and after one hour of PEC OER operation under an applied potential of 0.66 V_{RHE}. After PEC OER operation, the crystal structure of monoclinic $BiVO_4$ showed no significant change. The Bi 4f and V 2p XPS spectra exhibited no obvious variation, while the O 1s XPS spectra showed a decreased contribution from OH^- species. The Co 2p and Mn 2p XPS spectra displayed slight shifts in binding energy after operation. In addition, no obvious morphological change was observed for BVO_CMOH-2m in the SEM images. The CMOH layer remained on the surface of $BiVO_4$, although its uniformity changed slightly after operation. These results indicate that the CMOH coating imparted anti-photocorrosion behavior to $BiVO_4$. The CMOH layer also served as an OEC during PEC OER, which may have resulted in slight consumption and reconstruction of the overlayer.

Table S1. Summary of the $BiVO_4$ photoanode performance by various OEC decoration methods in PEC water oxidation.

Type of OECs	Post-treatment method<br>Spend time	Photocurrent density<br>(at 1.23 V_{RHE})	ABPE	η_{trans}<br>(at 1.23V_{RHE})	Ref.
CMOH	ARD process<br>1-16 min	6.0 mA /cm^2	1.68 %<br>at 0.66 V_{RHE}	81.7%	This work
MoOx/NiFe-LDH	Two step electrodeposition /Annealing<br>5~6 h	2.7 mA /cm^2	1.03 %<br>at 0.63 V_{RHE}	95%	5
NiFe-LDH/$Co_3Ge_2O_5(OH)_4$	Electrodeposition /Hydrothermal synthesis/Surface decoration<br>~50 h	5.15 mA/cm^2	1.85 %<br>at 0.63 V_{RHE}	87.2%	6
NiFeOx	Electrostatic adsorption/Calcination<br>~12 h	3.57 mA/cm^2	1.07 %<br>at ~0.76 V_{RHE}	77.8 %	7
Co_2AlO_4/CuCo-LDH	Two step chemical bath deposition/Annealing<br>4~5 h	2.69 mA/cm^2	1.57 %<br>at ~0.82 V_{RHE}	84.8%	8
CoMo-LDHs	Hydrothermal<br>~14 h	4.3 mA/cm^2	0.73 %<br>at 0.89 V_{RHE}	68.0 %	9
ultrathin-CoAl-LDH	Stirring/Dipping coating<br>~12 h	5.8 mA/cm^2	1.87 %<br>at 0.73 V_{RHE}	~97%<br>Nearly 100%	10
$Ru_{0.51}$-CoFe-LDH	Powder synthesis /Single-atom anchoring/Spin-coated loading<br>~72 h	4.51 mA/cm^2	1.55 %<br>at 0.66 V_{RHE}	76.0%	11
Co_3O_4/NiFe-LDH	Two step chemical bath deposition/	5.41 mA/cm^2	1.08 %<br>at 0.86 V_{RHE}	83.0 %	12

	Vacuum drying/ Calcination<br><br>~6 h				
NiCo-LDH modified Co-MOF-drived Co_3O_4	In situ MOF derivatization/ Calcination/LDH electrodeposition<br><br>~5 h	3.02 mA/cm^2	0.61 %<br><br>at 0.86 V_{RHE}	75.82 %	13
$CoFe_2O_4$/CoAl-LDH	Two step CBD/Calcination<br><br>~5 h	4.53 mA/cm^2	1.03 %<br><br>at 0.83 V_{RHE}	78.61%	14

REFERENCES

(1) Lai, C.-C.; Chen, J.-W.; Chang, J.-C.; Kuo, C.-Y.; Liu, Y.-C.; Yang, J.-C.; Hsieh, Y.-T.; Tseng, S.-W.; Pu, Y.-C. Two-Step Process of A Crystal Facet-Modulated $BiVO_4$ Photoanode for Efficiency Improvement in Photoelectrochemical Hydrogen Evolution. *ACS Appl. Mater. Interfaces.* **2022**, *14* (21), 24919–24928. DOI: 10.1021/acsami.2c03514.

(2) Devi, Y.; Huang, P.-J.; Chen, W.-T.; Jhang, R.-H.; Chen, C.-H. Roll-to-Roll Production of Electrocatalysts Achieving High-Current Alkaline Water Splitting. *ACS Appl. Mater. Interfaces.* **2023**, *15* (7), 9231–9239. DOI: 10.1021/acsami.2c19710.

(3) Wu, L.; Guan, S.; Zhou, B.; Guo, S.; Wang, J.; Wu, L.; Melvin, G. J. H.; Ortiz-Medina, J.; Wang, M.; Ogata, H. Plasma-Induced N Doping and Carbon Vacancies in A Self-Supporting 3C-SiC Photoanode for Efficient Photoelectrochemical Water Oxidation. *J. Mater. Chem. A* **2024**, *12* (30), 19201–19211. DOI: 10.1039/D4TA02612H.

(4) Dotan, H.; Mathews, N.; Hisatomi, T.; Grätzel, M.; Rothschild, A. On The Solar to Hydrogen Conversion Efficiency of Photoelectrodes for Water sSplitting. *J. Phys. Chem. Lett.* **2014**, *5* (19), 3330–3334. DOI: 10.1021/jz501716g.

(5) Ye, K.-H.; Li, H.; Huang, D.; Xiao, S.; Qiu, W.; Li, M.; Hu, Y.; Mai, W.; Ji, H.; Yang, S. Enhancing Photoelectrochemical Water Splitting by Combining Work Function Tuning and Heterojunction Engineering. *Nat. Commun.* **2019**, *10* (1), 3687. DOI: 10.1038/s41467-019-11586-y.

(6) Antuch, M.; Millet, P.; Iwase, A.; Kudo, A. The Role of Surface States during Photocurrent Switching: Intensity Modulated Photocurrent Spectroscopy Analysis of $BiVO_4$ Photoelectrodes. *Appl. Catal. B Environ.* **2018**, *237*, 401–408. DOI: 10.1016/j.apcatb.2018.05.011.

(7) Kang, B.; Hussain, M. B.; Cheng, X.; Peng, C.; Wang, Z. Green Electrodeposition Synthesis of NiFe-LDH/MoO_x/$BiVO_4$ for Efficient Photoelectrochemical Water Splitting. *J. Colloid Interface Sci.* **2022**, *626*, 146–155. DOI: 10.1016/j.jcis.2022.06.095.

(8) Chi, J.; Wei, Z.; Guo, W.; Fang, W.; Yan, J.; Huang, H.; Zhang, Y.; Luo, H.; Wang, J.; Liu, J. Enhanced Photoelectrochemical Water Splitting on $BiVO_4$ Photoanode via Efficient Hole Transport Layers of NiFe-LDH. *ACS Catal.* **2025**, *15*, 11293–11306. DOI: 10.1021/acscatal.5c02714.

(9) Xu, D.; Gao, X.; Gui, Z.; Duan, Y.; Li, Y.; Meng, X.; Gao, N.; Shi, W. Enhancement of $BiVO_4$ Photoanode Surface Oxygen Evolution Kinetics via Ni-Fe-ZIF Derived Bimetallic $NiFeO_x$ Co-catalyst for Water Oxidation. *Chem. Eng. Sci.* **2025**, *303*, 120965. DOI: 10.1016/j.ces.2024.120965.

(10) Wang, B.; Liu, Z.; Zhang, B.; Chen, M.; Lv, F.; Dong, Z. Dual Built-in Electrical field Constructed in Ternary $BiVO_4$/Co_2AlO_4/CuCo-LDH Photoanode for Boosted PEC Water Splitting. *Electrochim. Acta* **2026**, 148238. DOI: 10.1016/j.electacta.2026.148238.

(11) Jing, M.; Wang, Y.; Shi, G.; Li, L.; Wu, Y.; Liu, F.; Guo, A.; Yi, H. CoMo-LDHs Two-Dimensional Nanosheets Grown in $BiVO_4$ and Enhanced Photoelectrochemical Water Oxidation. *Int. J. Hydrogen Energy* **2025**, *120*, 473–485. DOI: 10.1016/j.ijhydene.2025.03.336.

(12) Zhong, Y.; Wu, C.; Jia, X.; Sun, S.; Chen, D.; Yao, W.; Ding, H.; Zhang, J.; Ma, T. Coupling of Self-Healing Atomic Layer CoAl-LDH onto Mo: $BiVO_4$ Photoanode for Fast Surface Charge Transfer toward Stable and High-Performance Water Splitting. *Chem. Eng. J.* **2023**, *465*, 142893. DOI: 10.1016/j.cej.2023.142893.

(13) Deng, W.; He, G.; Zhou, H.; He, W.; Gan, L.; Zhang, C.; Wang, K.; Qiu, X.; Liu, Y.; Li, W. Single-Atom Ru in CoFe-LDH Drives Efficient Charge Separation on $BiVO_4$ for Solar Water Splitting. *Nano-Micro Lett.* **2026**, *18* (1), 212. DOI: 10.1007/s40820-025-02062-y.

(14) Fu, Z.; Zhu, Y.; Qin, D.; Yang, Y.; Han, S.; Dong, Z. Functional Coupling Hole Transport and Extraction Units over $BiVO_4$ for Efficient Solar-to-Hydrogen Conversion. *Energy Convers. Manag.* **2024**, *322*, 119153. DOI: 10.1016/j.enconman.2024.119153.

(15) Jia, S.; Jiang, C.; Tian, K.; Bai, S.; Li, D.; Feng, Y.; Luo, R.; Chen, A. Energy Band-Matched Design of Co-MOF-Derived $Co_3O_4/BiVO_4$ Heterojunction Photoanode Synergised with NiCo-LDH to Improve Water Oxidation Performance. *J. Alloys Compd.* **2025**, 183388. DOI: 10.1016/j.jallcom.2025.183388.

(16) Dong, Z.; Chen, M.; Yu, M.; Liu, Z.; Zhang, B.; Han, S. Hetero-Interface Engineering of Ternary $BiVO_4/CoFe_2O_4$/CoAl-LDH Photoanode with Fast Charge Separation-Transfer for Boosted Photoelectrochemical Water Splitting. *Electrochim. Acta* **2026**, 148311. DOI: 10.1016/j.electacta.2026.148311.
